# Operations for Appendicitis

**Published:** 1909-08-14

**Authors:** 


### Surgery.

### OPERATIONS FOR APPENDICITIS.

In this article it is intended to compare some of the smaller differences in the methods adopted by various surgeons for the removal of the appendix and to discuss their relative merits. First, as to the site of incision in quiescent cases, the so-called "interval appendicectomies," the organ being removed in the interval between the two attacks. The two incisions most in vogue are an oblique one at McBurney's spot and a vertical one at the outer edge of the right rectus abdominis, the muscle being displaced inwards. Before the method of splitting the muscles of the abdominal wall in the direction of their fibres was introduced, there was little to choose between the two. But nowadays such good results are obtained by this method that in the writer's opinion it is the ideal way of opening the abdomen for appendicectomy. It may be claimed for it that a more immediate access to the appendix is given, and in a suitable case, *i.e.* if the abdominal wall is not covered with much fat and the muscles are not hypertrophied, the whole operation can be performed through an incision which is not longer than one and a half inches. One is usually taught to make the incision at the junction of the outer and middle thirds and a line which joins the umbilicus and the right anterior superior iliac spine, the line of the incision being at right angles to this. But it must be remembered that abdomens vary, the outer edge of the rectus being placed further out in some patients than in others. The edge of the rectus can nearly always be defined, and the writer thinks that it is, therefore, better to make an incision in the aforesaid line half way between the edge of the rectus and the iliac spine. This will expose the aponeurosis of the external oblique, its fibres lying parallel to the incision. These are separated and the internal oblique and transversalis are next seen lying at right angles to the line of the incision. These are similarly separated and held aside with retractors by an assistant, a manœuvre which gives a rectangular exposure through which the peritoneum can be incised and the cæcum and appendix easily brought to the surface. At the end of the operation the wound is sewn up in layers. It is almost impossible for a hernia to result, since the normal structure of the abdominal wall has hardly been deranged at all.

The vertical incision is necessarily longer than the oblique one, since it does not lie immediately over the appendix, and a larger exposure must therefore be obtained. The incision should be made just internal to the outer edge of the rectus and the muscle displaced inwards, after incision of the anterior wall of its sheath. The wound is sewn up in layers, and it follows that when the rectus falls back into its place the opening is closed by a sort of valvular apparatus, so that a hernia is unlikely to ensue. This is a much better method than going through the fibres of the rectus muscle itself.

The disadvantages of the method are the size of the wound that is necessary, and the fact that there may be some difficulty in bringing the appendix into the field of operation, especially if it lies retrocæcally. On the other hand, it is of extreme value in cases in which the diagnosis is not absolute; and this is often the case in women.

The great advantage of the vertical incision in such a case is that it can be easily enlarged downwards, so as to expose the pelvic viscera, while this cannot be done in the muscle-splitting operation.

Secondly, as to the actual method of dealing with the stump of the appendix after cutting the meso-appendix and ligaturing the vessels in it. Here again there are many small differences of technique among surgeons. The commonest method is to turn down a collar of the peritoneal coat and to bring it together over the base of the appendix. This takes a little time, and the writer prefers simply to ligature the appendix, serous coat and all, at its base, and then to invaginate the stump with two or three Lembert sutures, which pass through the serous and muscular coat of the cæcum. All that is claimed for this is that it can be done in less time than it takes to turn down a peritoneal cuff. After all, the great point is to be sure that the entire appendix has been removed.

Whichever method is adopted the stump of the appendix is shut off in peritoneal surroundings, and it therefore seems unnecessary to touch the stump with pure carbolic acid or any other powerful antiseptic. Quiescent cases do uniformly well without this, and on general principles it is inexpedient to introduce strong chemicals into the peritoneal cavity.

Coming to the operation in the acute stage, when an abscess is present, many surgeons make a great point of packing off the rest of the peritoneal cavity with gauze before opening the abscess, the idea being that if any pus were allowed to escape general peritonitis would supervene. But even if pus did escape it is unlikely that this would ensue, since the peritoneum has already walled off one abscess, and is therefore in a high state of resistance to the bacillus coli. It cannot be said that this packing with gauze does any harm, but it is difficult to see how it does any good; because it is a practical impossibility to shut off the peritoneum so completely by this means that not a single drop of pus can escape. All that is necessary is to break down the wall of the abscess carefully and remove the pus as it escapes by means of a series of swabs held in forceps. Too little faith is reposed in the natural power of the peritoneum to resist infection. It is really the best friend of the surgeon. The same argument can be used against those who hold that it is highly dangerous to search for an appendix in an abscess cavity lest the wall of the cavity is ruptured and peritonitis follow. Peritonitis will not follow in a peritoneum which is in a high state of reaction. The worst that can happen is that a second remote abscess may form; and this is almost preferable to leaving a diseased appendix in the abdomen.

